# An Efficient DenseNet-Based Deep Learning Model for Malware Detection

**DOI:** 10.3390/e23030344

**Published:** 2021-03-15

**Authors:** Jeyaprakash Hemalatha, S. Abijah Roseline, Subbiah Geetha, Seifedine Kadry, Robertas Damaševičius

**Affiliations:** 1 Department of Computer Science and Engineering, AAA College of Engineering and Technology, Sivakasi 626123, Tamil Nadu, India; jhemalathakumar@gmail.com; 2 School of Computer Science and Engineering, Vellore Institute of Technology—Chennai Campus, Vandalur—Kelambakkam Road, Chennai 600127, Tamil Nadu, India; abijahroseline.s2017@vitstudent.ac.in (S.A.R.); geetha.s@vit.ac.in (S.G.); 3 Faculty of Applied Computing and Technology (FACT), Noroff University College, 4608 Kristiansand, Norway; skadry@gmail.com; 4 Faculty of Applied Mathematics, Silesian University of Technology, 44-100 Gliwice, Poland

**Keywords:** malware detection, malware visualization, cybersecurity, densely connected convolutional network, deep learning

## Abstract

Recently, there has been a huge rise in malware growth, which creates a significant security threat to organizations and individuals. Despite the incessant efforts of cybersecurity research to defend against malware threats, malware developers discover new ways to evade these defense techniques. Traditional static and dynamic analysis methods are ineffective in identifying new malware and pose high overhead in terms of memory and time. Typical machine learning approaches that train a classifier based on handcrafted features are also not sufficiently potent against these evasive techniques and require more efforts due to feature-engineering. Recent malware detectors indicate performance degradation due to class imbalance in malware datasets. To resolve these challenges, this work adopts a visualization-based method, where malware binaries are depicted as two-dimensional images and classified by a deep learning model. We propose an efficient malware detection system based on deep learning. The system uses a reweighted class-balanced loss function in the final classification layer of the DenseNet model to achieve significant performance improvements in classifying malware by handling imbalanced data issues. Comprehensive experiments performed on four benchmark malware datasets show that the proposed approach can detect new malware samples with higher accuracy (98.23% for the Malimg dataset, 98.46% for the BIG 2015 dataset, 98.21% for the MaleVis dataset, and 89.48% for the unseen Malicia dataset) and reduced false-positive rates when compared with conventional malware mitigation techniques while maintaining low computational time. The proposed malware detection solution is also reliable and effective against obfuscation attacks.

## 1. Introduction

The increasing number and complexity of malware have become one of the most serious cybersecurity threats [1,2]. Although the cybersecurity industry is constantly working to monitor and thrive in several ways with malware, cyber attackers show no indications of stopping or slowing down their attacks. Malicious hacker groups develop sophisticated evasive malware techniques such as polymorphism [3], metamorphism [4], code obfuscations [5], etc., that outperform many traditional malware mitigation systems. The most widely used malware by attackers targeting businesses are backdoors, miners, spyware, and information stealers. Emotet [6] and TrickBot [7] are information stealers that commonly use malicious spam (malspam) to infect systems. Malspam contains infected attachments or URLs. These malware variants can gather sensitive information from organizations. Other examples of recent malware include WannaCry, Kovter, ZeuS, Dridex, IcedID, Gh0st, Mirai, etc. The design of efficient malware detection systems is still in an arms race between the attackers and the security analysts.

Malware cases are analyzed using static and dynamic methods [8]. The code logic is analyzed or disassembled without its execution in a static analysis method. They extract features such as opcodes, Application Programming Interface (API) sequences, system calls, etc. In dynamic analysis, the behavior of malware is analyzed by executing malignant code in a safe and controlled environment. This analysis extracts network activity, system calls, file operations, registry modifications, etc., as features. Static detection methods are ineffective in identifying new malware as the signatures are not generalized. Dynamic behavior-based methods improve detection accuracy, but they pose high overhead. Data mining methods require many training samples and training time. Traditional dynamic malware analysis methods are ineffective in the sense that they allow the malware to be executed in a controlled environment such as a virtual box or vmware. The malware is executed, and the various methods of its persistence mechanism, the way it is propagated, the damage it causes to a system, and the network are investigated in detail. This requires good expertise in the domain and a controlled environment for execution. As far as static analysis methods are concerned, portable executable (PE) files are completely disassembled and their hex codes are investigated to understand the flow and impact of the malware. This requires a great deal of expertise in assembly codes and a very thorough understanding of the malware and its operations. Additionally, it also requires memory as well as time. Though these methods are employed, combating new malware efficiently is becoming more difficult [9].

**Figure 1:**
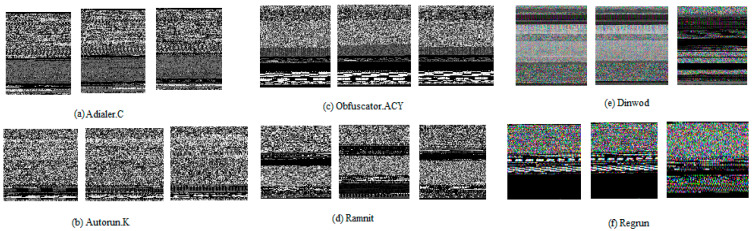
A sample of malware images belonging to various classes of the malware datasets (**a**) Adialer.C, (**b**) Autorun.K, (**c**) Obfuscator.ACY, (**d**) Ramnit, (**e**) Dinwold, and (**f**) Regrun.

Visualization of malware has been recently used as a new and efficient technique for malware research. Figure 1 shows the malware images of various classes of Malimg [10], Microsoft BIG 2015 [11], and MaleVis [12] datasets. The preexisting binary patterns are being substantially reused to achieve variations, generating new patterns. This means that malware samples can be grouped into several families and that each variant possesses its respective family characteristics. Hence, it is critical to detect malware efficiently and to identify its variations.

Motivated by the work of Nataraj et al. [10], we view a malware detection problem as a multi-class image classification problem by visualizing a binary code into a two-dimensional (2D) grayscale image. The structure of the PE binary file (cleanware or malware) is studied by converting it into an image to provide more information about it. The binary images corresponding to the same class appear quite similar in structure and texture, where they are distinct between different classes. The various subsections of a PE binary are visualized with different textures. The small modifications made to the binary by malware writers are recognizable in new variants, but the overall structure of the image remains unaffected. Since it is crucial in detecting malware and to avoid information loss, no other approach for visualization is effective.

Deep learning is a subfield of machine learning, which learns the input at multiple levels to gain better knowledge representations. Advances in computer vision with deep learning were developed, mainly through Convolutional Neural Networks (CNN). Deep learning models learn complex features and train a complex model with many convolutional layers requiring millions of parameters. This eventually leads to overfitting in only a few epochs, and the model does not generalize well, resulting in poor model performance. The knowledge of CNNs thoroughly trained on a massive, well-elucidated dataset such as ImageNet [13] can also be transferred to make the detection and classification of malware images more effective. The key idea of transfer learning is that the knowledge gained in learning a model can help to enhance a different task in learning. CNNs are built on increasingly deeper and input passes through many layers. The input information may vanish before it reaches the final layer of the network. ResNet and other CNNs address this problem, but they generate shorter paths from preceding layers to the subsequent layers. 

In this paper, a novel method is presented to classify malware variants based on the deep learning DenseNet model [14] enhanced with a class-balanced loss for reweighting the categorical cross-entropy loss. The proposed modification of the DenseNet model ensures information flow by directly connecting all the layers with their feature maps in the network. The feedforward approach is maintained by acquiring additional inputs from the previous layers and passes on feature maps of the current layer to all succeeding layers. The proposed model was practically assessed using the TensorFlow Python library [15] and obtained promising results for analysis of the Malimg [10], Microsoft BIG 2015 [11], MaleVis [12], and Malicia [16] datasets.

The contribution of this work is as follows:

- An effective and expeditious deep learning-based malware detection and classification system using raw binary images while requiring no binary execution (behavioral analysis), reverse engineering, or code disassembly language skills is provided.
- The proposed methodology employs pretrained Densely Connected Convolutional Networks (DenseNet) to achieve faster preprocessing and training of binary samples. The DenseNet model allows for concatenation of features and utilizes fewer parameters compared to other CNN models. The implicit deep supervision mechanism of the DenseNet model contributes to effective malware detection. Additionally, the dense connections with its regularizing power help reduce overfitting with smaller malware training datasets.
- The data imbalance problem in classifying malware is tackled using reweighting of the class-balanced categorical cross-entropy loss function in the softmax layer.
- We conduct an extensive evaluation on four different malware datasets, of which three datasets are used for training and one dataset is used for testing the proposed model. The results show that the proposed system is very efficient and effective. It is also resilient against sophisticated malware evolution over time and against anti-malware evasion tactics.
- Without the need for complex feature engineering tasks, the proposed deep learning-based malware detection model achieves higher accuracy rates of 98.23%, 98.46%, and 98.21% for the three datasets and of 89.48% for the unseen (Malicia) dataset. The model has high computational performance, achieving an efficient malware detection system.

The paper is organized as follows. Section 2 presents a literature survey on malware recognition and classification. Section 3 details the proposed DenseNet-based malware detection system. Section 4 describes the malware datasets used to assess the performance of the proposed system. The experimental results of the proposed model and performance analysis with other known malware detection systems are also discussed. The conclusion of the paper is presented in Section 5.

## 2. Literature Survey

Significant malware analysis and detection research surveys have been conducted based on static, dynamic, and machine learning methods [17,18]. This section provides a survey of the different methods used to classify malware. Static features such as byte, string, and opcode sequences [19]; function length distribution [20]; functional call-graph [21]; and PE file features [22] are extracted using static analysis methods. Schultz et al. [23] obtained various static features from binary files and analyzed their performance by training with different machine learning techniques. Roseline and Geetha [24] used static features to classify malware using an oblique random forest approach. Common signature-based malware detection approaches include malicious code analysis, signature generation, and signature database storage. These approaches are inefficient since malware attackers execute malicious activities and constantly create zero-day malware. Static analysis is not resilient to code obfuscation and does not enable automated processing.

Behavioral features such as network activities, instruction sequences, and system calls [25] are extracted using dynamic analysis methods. Imran et al. [26] proposed a malware classification approach based on similarity. The API call sequences were obtained using Hidden Markov Models (HMMs), and similarity scores were computed for malware classification. Their approach works well with fewer data and requires high computation overhead. Dynamic analysis is inefficient as malware may modify its behavior in virtual environments during execution. Hybrid methods use features derived from static, dynamic, or machine learning methods [27] to classify malware. Rieck et al. [28] extracted the dynamic API call features and used Support Vector Machine (SVM) for detecting malware. Islam et al. [8] showed that the hybrid approach is more efficient than static or dynamic approaches.

Recently, significant research efforts in malware analysis made use of the vision-based approach [29,30,31,32,33]. Features such as opcode sequences and system calls were visualized as images [34,35]. Han et al. proposed an effective system for identifying packed and encrypted malware. Malware binaries were converted into images [36] for classifying their variants. Conti et al. [37] first reported that visual methods help researchers efficiently classify binary files, analyze new file structures, and obtain perspectives that impart knowledge and enhance the existing set of commonly used methods. The byteview visualization enabled the researchers to easily identify the presence of significant sections in the file. Nataraj et al. [10] extracted GIST texture features from visualized grayscale images and classified malware using K-Nearest Neighbors (KNN) with Euclidean distance. Their system required less computational cost than the n-gram method for malware classification. Han et al. [35] proposed an automatic analysis method for generating entropy graphs from grayscale images. Their method did not identify packed malware since the entropy measure was high and patterns were not visualized. Kancherla et al. [38] extracted Gabor, intensity, and wavelet features from binary images. Their approach was robust to code obfuscations. Liu et al. [39] presented an approach based on grayscale images, and the image size was reduced using the local mean method to achieve better ensembling. Fu et al. [40] visualized malware as RGB (Red, Green, Blue) color images and extracted global texture and color features from them. The code and data segments were also extracted as local features. Their method was a combination of taking global as well as local features, achieving effective malware classification. Nisa et al. [41] converted malware to images and applied segmentation-based fractal texture analysis (SFTA) to obtain features, which were fused with features obtained from pretrained AlexNet and Inception-v3 deep neural networks. Finally, machine learning classifiers were used for malware detection. Azab et al. [42] proposed a malware spectrogram image classification framework that uses spectrogram images classified by CNN for malware detection. Ding et al. [43] extracted bytecode from the Android package (APK) file and transformed it into a 2D bytecode matrix. Then, a CNN model was trained and used for malware recognition. Mahdavifar and Ghorbani [44] proposed a deep learning expert system that extracts refined rules from a trained deep neural network (DNN) model for malware detection. Naeem et al. [45] converted APK files to color images and used a convolutional DNN to extract dynamic image features. Then, the DNN was trained to detect malware attacks. Singh et al. [46] proposed a methodology to convert malware features into fingerprint images. Then, a CNN model was used to extract features from visualized malware for malware recognition. Sun and Qian [47] generated malware feature images by aggregating static analysis of malware using recurrent neural network (RNN) and CNN models. Feature images were obtained by fusing original codes with predictive codes obtained from RNN. Finally, a CNN was trained to recognize malware.

Previous works based on traditional methods are time-consuming and inefficient with the growing amount of malware. The visualization method is effective in terms of time and processing efficiency. Conventional machine learning methods are not able to handle raw pixel information from images and do not enable incremental learning. The transformation of raw data into feature vectors needs extensive engineering and technical knowledge. The classification model trains the transformed form of input images. Deep learning techniques achieve this representational learning ability to use raw input data and allow for automated learning.

Deep learning techniques [48] are focused on multiple layers of abstraction, with higher layers representing more abstract data information. Neural networks replace typical machine learning techniques as an alternative in detecting malware. The advantages of neural network models include incremental learning ability, training layers as required, etc. Deep learning contributes to the development of automated and generalized models for the detection and classification of known and unknown malware [49]. CNNs are feed-forward neural networks specifically used for image classification problems. Considering the ability of robust feature learning, state-of-the-art malware detection systems use CNN models [50] for learning binary patterns in malware images. Ensemble models [51,52,53] can combine multiple machine learning and deep learning models using stacking, boosting, or bagging architecture. Cui et al. [54] proposed a CNN model for malware detection. Their system worked for input images of fixed sizes. Agarap et al. [55] trained the hybrid combination of deep learning models and SVM on the Malimg dataset. Their approach provided insights into designing an intelligent malware detection system. The proposed model analyzes malware based on the vision-based technique. The advantages of the CNN model are considered to train the malware images and to effectively classify them using the proposed modification of the DenseNet model.

## 3. Proposed Methodology 

**Figure 2:**
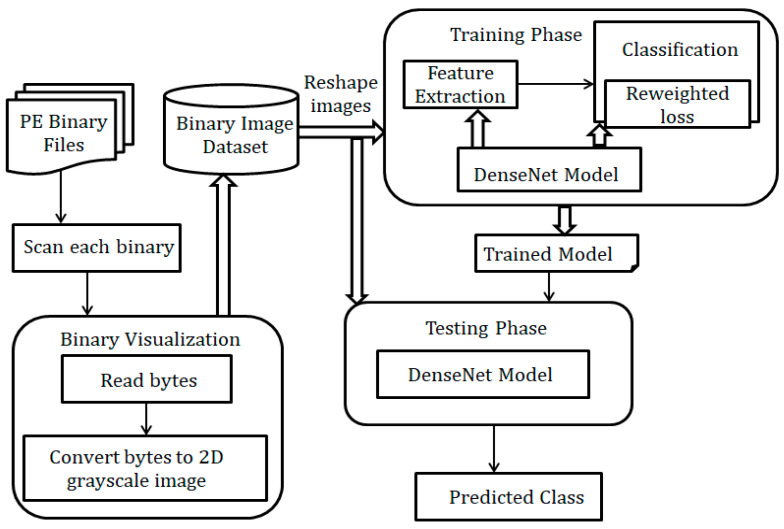
Structural diagram of the proposed model.

**Figure 3:**
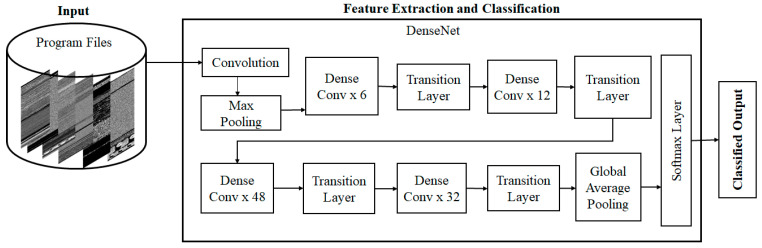
Flow of the DenseNet Model.

The overall design of the proposed malware detection approach is illustrated in Figure 2. The flow of the proposed modification of DenseNet model is shown in Figure 3. The input binary images are fed into the DenseNet model for feature extraction and classification. The model is trained by providing the input image directly into the initial convolution (Conv) layer. CNNs have a great potential to extract distinctive features that comprehensively articulate the image and learn task-specific features. They automatically learn features at various levels of abstraction, allowing them to learn complex functions by modeling raw input data into the desired output. The proposed model uses DenseNet to extract all features from malware datasets and trains the DenseNet on top of the extracted features. Every dense layer can extract fine details from binary images.

The proposed model was built with an initial convolutional layer, max-pooling layer, four dense convolution (Dense Conv) blocks, and four transition layers (1×1 Conv and 2×2 average pooling). Dense Conv blocks consist of a collection of 1×1 Conv and 3×3 Conv blocks. After each alternative transition layer, these convolutions (1×1 and 3×3) are repeated 6, 12, 48, and 32 times within each Dense Conv block. The output feature maps obtained after passing through these layers are given as an input for the Global Average Pooling (GAP) block. Next, a fully connected (FC) layer follows GAP. The FC layer classifies the malware samples into their corresponding classes. 

### 3.1. Preprocessing of Input Binaries

The PE binary files are read as bytes in the range 0 to 255 and stored as a one-dimensional (1D) vector of 8-bit unsigned integers. Each byte represents the pixel intensity levels (0 denotes black, 255 denotes white, and intermediate values denote various gray shades). These byte values are organized into a two-dimensional array (pixels), which are visualized as grayscale images. The sizes of the binary files are of variable sizes originally. The CNNs do not accept images of different resolutions, since it is composed of the FC layers with a fixed number of trained weights. Therefore, the input images of various dimensions are resized into a square image of 64 × 64 dimensions. The images are resampled using the nearest interpolation method since it does not alter the actual image data. It chooses the value of the pixel that is close to the neighboring coordinates of the desired interpolation point. This method locates the closest pixel in the original input image for each pixel in the resulting image. The nearest interpolation approach is beneficial over other interpolation methods, such as bilinear and bicubic interpolation, in terms of its simplicity, its capability to retain original values in the unalterable setting, as well as its computational time. This approach is used in our work since malware images should not be changed and critical information should not be lost to provide accurate resampling.

### 3.2. DenseNet

DenseNet [14] is a deep learning architecture in which all layers are directly connected, thereby achieving effective information flow between them. Each layer acquires additional inputs from all previous layers and transfers its feature maps to all subsequent layers. The output feature maps obtained from the current layer are combined with the previous layer using concatenation. Every layer is linked with all the succeeding layers of the network, and they are referred to as DenseNets. This model requires fewer parameters compared to traditional CNNs. It also reduces the overfitting problem that occurs with smaller malware training sets.

Consider an input image $x_{0}$, which is passed through the proposed convolutional network. The network contains N layers, and each layer executes a nonlinear transformation $F_{n}\left(.\right)$. Suppose that layer n consists of the feature maps of all preceding convolutional layers. The input feature maps of layers 0 to n−1 are concatenated and represented as $x_{0},\ldots ,x_{n-1}$. Hence, this model has N(N+1)/2 connections on an N-layer network. The output of the *n*th layer is given by

(1) $$x_{n}=\;F_{n}\left(\left[x_{0},\ldots ,x_{n-1}\right]\right),$$

where $x_{n}$ is the current *n*th layer, $\left[x_{0},\ldots ,x_{n-1}\right]$ is a concatenation of feature maps obtained from 0 to *n* − 1 layers, and $F_{n}\left(.\right)$ is the composite function of Batch Normalization (BN)-Rectified Linear Units (ReLU).

The consecutive operations in the transition layer include Batch Normalization (BN), Rectified Linear Units (ReLU), and 3 × 3 convolution (Conv). The concatenation operation is not feasible if the sizes of feature maps are changed. Therefore, the layers that have different feature map sizes are downsampled. The transition layers consisting of 1×1 Conv and 2×2 average pooling operations are given between two adjacent Dense Conv blocks. The initial Conv layer consists of 7×7 Conv blocks with stride 2. After the final Dense Conv block, the classification layer consisting of global average pooling and the softmax classifier are connected. The correct prediction is done using all feature maps in the neural network. The output layer with K neurons gives the correct match of K malware families.

Convolution operation learns the image features and maintains the connection among the pixels. Mathematically, a convolution function operates on an image matrix and filter. Each convolution layer corresponds to the BN-ReLU-Conv sequence. After the convolution is performed on the image, ReLU is applied to the output feature maps. This function introduces nonlinearity in CNNs. The ReLU function is given by

(2) $$f\left(x_{0}\right)=\max\left(0,\;x_{0}\right).$$

Pooling is performed to reduce the dimensionality of output feature maps. This pooling is performed either using max pooling or average pooling. Max pooling involves taking the largest component from the improved feature map. Average pooling divides the input into the pooling area and computes the average values of each area. GAP computes the average of each feature map, and the resulting vector is taken to the softmax layer.

The operations of the proposed network are summarized in Algorithm 1.
**Algorithm 1.** **DenseNet algorithm.****Input:** PE binary files **Output:** Correct matching class *c_i_*1. Transform binaries to two—dimensional array grayscale images I, where $I\in \left\{x_{0},x_{1},\ldots ,x_{n}\right\}$, $x_{0},x_{1},\ldots ,x_{n}$—set of all input images. 2. Train the model.a. Extract raw features from the input image.b. Perform initial convolution and generate feature maps.c. Link each layer by concatenating the feature maps of all preceding layers.d. Perform 1 × 1 and 3 × 3 convolutions for 6 times in the first Dense Conv block.e. Perform 1 × 1 convolution with 2 × 2 average pooling in the first transition layer.f. Perform 1 × 1 and 3 × 3 convolutions 12 times in the second Dense Conv block.g. Perform 1 × 1 convolution with 2 × 2 average pooling in the second transition layer.h. Perform 1 × 1 and 3 × 3 convolutions for 48 times in the third Dense Conv block.i. Perform 1 × 1 convolution with 2 × 2 average pooling in the third transition layer.j. Perform 1 × 1 and 3 × 3 convolutions for 32 times in the fourth Dense Conv block.k. Perform 1 × 1 convolution with 2 × 2 average pooling in the fourth transition layer.l. Perform global average pooling at the end of step 2(k).3. Classify the input images into their respective classes using a softmax classifier.

### 3.3. Classification

The classification layer is composed of a fully connected softmax layer. In FC, the number of neurons is set according to the number of malware classes available in the dataset. The softmax function is used for categorizing multi-class classification problems. This function calculates the probability distributions of each class i over all possible classes. The softmax activation function is given by

(3) $$S\left(y_{i}\right)=\;\frac{e^{y_{i}}}{\sum_{j}e^{y_{j}}},$$

where $y_{i}$ is the input value and $y_{j}$ is all input values of I. The formula calculates the ratio of the exponential of the input element and the sum of the exponential values of all input data. 

The class imbalance problem is a classification challenge in which the distribution of classes in the training dataset is uneven. The degree of class imbalance varies, but a significant imbalance is more difficult to model and demands advanced techniques to tackle the issue. The Malimg dataset and the Microsoft BIG 2015 dataset are imbalanced and long-tailed malware datasets that contain more samples for few classes and very few samples in some classes. Models trained on these varied sample sizes are biased toward dominant classes. To resolve the issue of data imbalance, data augmentation techniques such as oversampling of minority classes or downsampling of majority classes are not appropriate for malware detection problems. It is not possible to generate images corresponding to realistic malware binaries by oversampling. Many representative malware variants might be possibly overlooked by downsampling. 

Reweighting losses by inverse class frequency typically results in low performance on real-world data with a high-class imbalance. The proposed malware detection model uses class-balanced loss [56] and uses a weighting factor $W_{i}$, which has an inverse ratio to the number of samples for class i. It is given by

(4) $$W_{i}\propto 1/S_{n_{i}},$$

where $S_{n_{i}}$ is the effective number of samples for class i. It is given by

(5) $$S_{n_{i}}=\left(1-B_{i}^{n_{i}})/(1-B_{i}\right),$$

where B=(I−1)/I and I is the set of all possible instances in a class, defined as

(6) $$I=\underset{n\to \infty }{\lim}\sum_{i=1}^{n}B^{i-1}=1/\left(1-B\right).$$

### 3.4. Training 

The Adaptive Learning Rate Optimization Algorithm, called Adam [57], is used to update weights based on the malware training data. It determines the individual learning rates for distinct parameters. Adam uses evaluations of the 1st and 2nd moments of the gradient to adjust the learning rate for individual weights of the neural network. Therefore, it is known as adaptive moment estimation. This optimizer evaluates the moments using increased moving averages. These moving averages are based on the calculated gradient on the current mini-batch. The moving average estimates of the first and second moments of the gradient are given by

(7) $$a_{t}=\;\beta_{1}a_{t-1}+\left(1-\;\beta_{1}\right)\;g_{t},$$

(8) $$b_{t}=\;\beta_{2}b_{t-1}+\left(1-\;\beta_{2}\right)\;g_{t}^{2},$$

where a is the moving average, $\beta_{1}$ and $\;\beta_{2}$ are decay rates, and g is the gradient on the current mini-batch.

Cross-entropy (*CE*) loss or log loss assesses the efficiency of a classification method with a probability score between 0 and 1. When the predicted probability deviates from the real class label, the *CE* loss increases. The cross-entropy loss is given by

(9) $$CE=\;-\sum_{i}^{C}t_{i}\;\log\left(s_{i}\right),$$

where *C* is the set of all classes in each dataset, $t_{i}$ is the ground truth, and $s_{i}$ is the CNN score for each class i in C.

Categorical *CE* loss is a combination of softmax activation function and *CE* loss, also known as Softmax Loss, used for multiclass classification. It outputs a probability value for each input binary image over C.

The Categorical Cross-Entropy (*CCE*) loss for a sample s corresponding to class label y is given by

(10) $$CE\left(f,y\right)=-\log\left(\frac{\exp\left(f_{y}\right)}{\sum_{i=1}^{C}\exp\left(f_{i}\right)}\right).$$

The Class Balanced Cross-Entropy (*CBCE*) loss for class y with $n_{y}$ training samples is given by

(11) $$CBCE\left(f,y\right)=-\frac{1-B}{1-B^{n_{y}}}\log\left(\frac{\exp\left(f_{y}\right)}{\sum_{i=1}^{C}\exp\left(f_{i}\right)}\right).$$

## 4. Experimental Results

### 4.1. Datasets

The proposed model was evaluated with four malware datasets: Malimg [10], Microsoft’s BIG 2015 [11], MaleVis [12], and Malicia [16]. The first three datasets were used for training, and the fourth (Malicia) dataset was used for testing. The experiments were carried out with 1043 cleanware samples. These samples were collected from executable files (.exe) of the Windows operating system and checked using the VirusTotal portal. The various families of the malware datasets used for evaluation of the proposed malware detection method are given in Table 1. The samples of different classes of malware vary in number across different datasets. There were 9339 malicious samples presented as grayscale images in the Malimg dataset. Each of the malware samples in the dataset corresponds to one of the 25 malware classes.

The BIG 2015 dataset contains 21,741 malware samples, among which the training set includes 10,868 samples and the remaining 10,873 samples are test samples. In our experiments, the training set samples are used for evaluation. Each malware file has an identifier and class. The identifier is a hash value that particularly identifies the file, while the class labels one of nine distinct malware families. Each malware has two files, namely, .bytes and .asm. We use .bytes files, which have raw hexadecimal code of the file, to generate malware images. 

**Figure 4:**
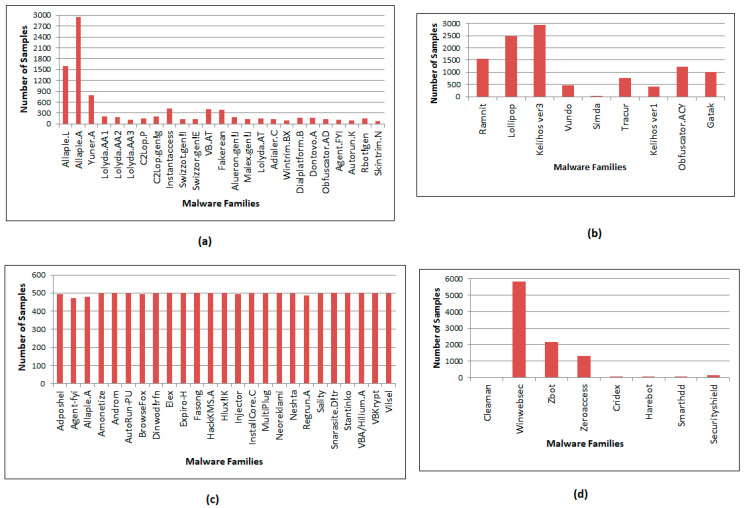
Distribution of malware over classes in the (**a**) Malimg, (**b**) BIG 2015, (**c**) MaleVis, and (**d**) Malicia datasets.

The MaleVis dataset consists of 14,226 RGB byte images assigned to one of 26 families (25 malware + 1 cleanware). The Malicia dataset includes 8 classes consisting of 9670 malware samples. The dataset is untrained by the proposed DenseNet model to evaluate how well it performs under different samples. The three trained datasets contain completely different classes from the Malicia dataset classes. Figure 4 illustrates the distribution of samples over classes for all four datasets. 

### 4.2. Results and Discussion

The dataset was randomly divided into 70% training and 30% validation sets. The results were taken with 1043 cleanware samples and each of the three malware datasets. Train and test files were divided such that 30% of the overall samples were considered for testing purposes. The proposed malware detection system was trained on 7268 samples and tested on 3115 samples for the Malimg dataset with cleanware samples (9339 + 1043). Then, the model was trained on 8338 samples and tested on 3573 samples from the BIG 2015 dataset along with cleanware samples (10,868 + 1043). On the MaleVis dataset, 9958 samples were training samples and 4268 were testing samples.

The experiments were implemented on a Linux system with Intel^®^ Xeon(R) CPU E3-1226 v3 at 3.30 GHz × 4, 32 GB RAM, and NVIDIA GM107GL Quadro K2200/PCIe/SSE2. The performance evaluations were carried out with the following hyperparameter settings: 100 epochs, learning rate 0.0001, and batch size 32. The proposed deep neural network model was implemented on the Python framework and Keras v0.1.1 deep learning library. The experiments were performed for various input binary image sizes such as 32 × 32 dimensions and 64 × 64 dimensions. It is observed that the information is retained and showed better predictive accuracy for images reshaped to 64 × 64.

There are four types of metrics calculated to assess classification predictions.

True Positive (TP): the prediction that an observation belongs to a class and it actually does belong to that class, i.e., a binary image that is classified as malware and is actually malware.

True Negative (TN): the prediction that an observation does not belong to a class and it actually does not belong to that class, i.e., a binary image that is classified as not malware (negative) and is actually not malware (negative).

False Positive (FP): the prediction that an observation belongs to a class and it actually does not belong to that class, i.e., a binary image that is classified as malware and is actually not malware (negative).

False Negative (FN): the prediction that an observation does not belong to a class and it actually does belong to that class, i.e., a binary image that is classified as not malware (negative) and is actually malware.

These four outcomes are presented on a confusion matrix to better describe the results of the proposed model. If there are N classes, the confusion matrix will be the N×N matrix, with the true class on the left axis and the class assigned to an element with that true class on the top axis. Each member a,b of the matrix is the number of elements with actual class a that is classified as belonging to class b.

The elements of confusion matrix for each class are defined by

$${\mathrm{TP}}_{a}=C_{\mathrm{aa}}$$

$${\mathrm{FP}}_{a}=\sum_{i=1}^{n}C_{\mathrm{ia}}-{\mathrm{TP}}_{a}$$

$${\mathrm{FN}}_{a}=\sum_{i=1}^{n}C_{\mathrm{ai}}-{\mathrm{TP}}_{a}$$

$${\mathrm{TN}}_{a}=\sum_{i=1}^{n}\sum_{j=1}^{n}C_{\mathrm{ij}}-{\mathrm{TP}}_{a}-{\mathrm{FP}}_{a}-{\mathrm{FN}}_{a}$$

Accuracy (Acc), Precision (Pr), Recall (Re), and F1 score are the four main classification metrics. The number of correct predictions divided by the total number of predictions is known as accuracy. It is defined as

$$\mathrm{Acc}=\frac{\mathrm{TP}+\mathrm{TN}}{\mathrm{TP}+\mathrm{TN}+\mathrm{FP}+\mathrm{FN}}$$

Precision is the number of correct positive outcomes divided by the number of positive outcomes predicted by the classifier.

$$\mathrm{Pr}=\frac{\mathrm{TP}}{\mathrm{TP}+\mathrm{FP}}$$

Recall gives the fraction of correctly identified instances as the positive out of all positives.

Re=TPTP+FN

F1 score is the harmonic mean of precision and recall. It determines the classifier’s precision (the number of instances it correctly classifies) as well as its robustness (it does not miss a substantial number of instances). It is given by

$$F1\;\mathrm{score}=2\times \frac{\mathrm{Precision}\times \mathrm{Recall}}{\mathrm{Precision}+\mathrm{Recall}}.$$

The comparison results of Machine Learning (ML) and Deep Learning (DL) methods for malware detection are presented in Table 2 and Table 3, respectively. The performance analysis of the proposed model is compared with various ML techniques such as K-Nearest Neighbor (KNN), Logistic Regression (LR), Naïve Bayes (NB), SVM, Decision Tree (DT), Random Forest (RF), and Adaboost. The malware detectors based on pretrained DL models such as CNN and its variants are used for analyzing the efficiency of the proposed DenseNet-based malware detection method. The performance results obtained for the proposed model are better than the ML and DL-based malware detection models for the three datasets. The proposed model obtained an accuracy of 98.23% for Malimg, of 98.46% for BIG 2015, and of 98.21% for MaleVis dataset. 

The generalization ability of the proposed method is assessed using unseen dataset. The dataset is untrained by the proposed DenseNet model to evaluate how well it performs under different samples. The three trained malware datasets contain completely different classes from the Malicia dataset classes. The comparison of the proposed methods with the ML and DL methods over the unseen Malicia dataset is given in Table 4. The results on the unseen Malicia dataset show an accuracy of 89.48%, which is less than the performances of the ML and DL methods over the trained datasets. 

Table 5 provides details about the time taken for the proposed model to train and test the binary samples. The comparison of the proposed model and the malware detectors based on various DL methods are studied in terms of computational efficiency. The results indicate that the proposed DenseNet-based malware detection model takes less time to train and test the samples when compared to other deep learning-based malware detection systems.

Table 6 compares the results of the proposed malware detection model with previous works on the four malware datasets (3 training dataset + 1 (unseen) test dataset). The proposed model outperforms other detection methods in the literature. The accuracy of the proposed model (98.23%) is slightly higher than the accuracy of the method by Roseline et al. (98.65%) on the Malimg dataset. The results of the proposed model outperform the existing methods on the BIG 2015, MaleVis, and Malicia datasets.

**Figure 5:**
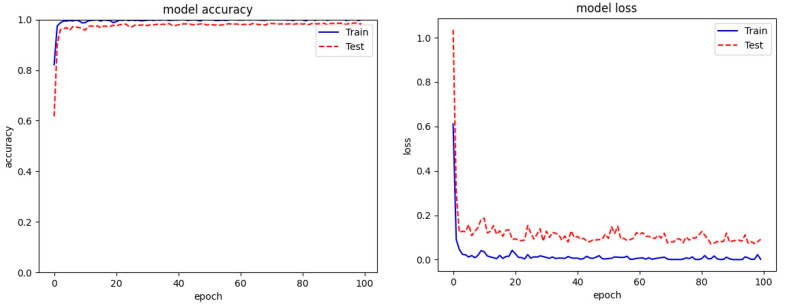
Training and test accuracy and loss for the Malimg dataset.

**Figure 6:**
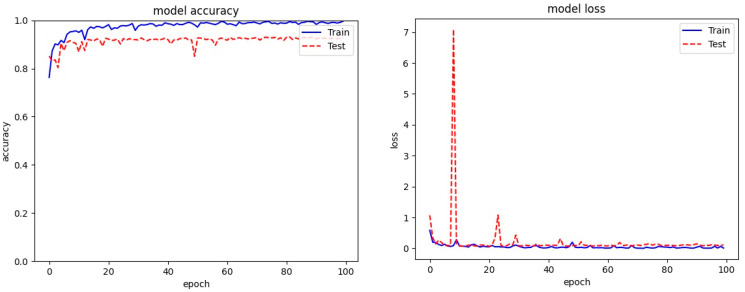
Training and test accuracy and loss for the BIG2015 dataset.

**Figure 7:**
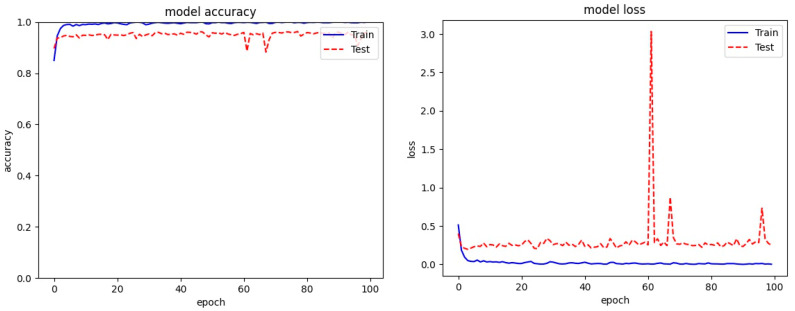
Training and test accuracy and loss for the MaleVis dataset.

Figure 5, Figure 6 and Figure 7 present the plots for train accuracy, test accuracy, and loss over the number of epochs for the proposed model with the Malimg, BIG 2015, and MaleVis datasets. From the figures, the accuracy is observed as rising for increasing epochs and the loss decreases as epochs increase.

**Figure 8:**
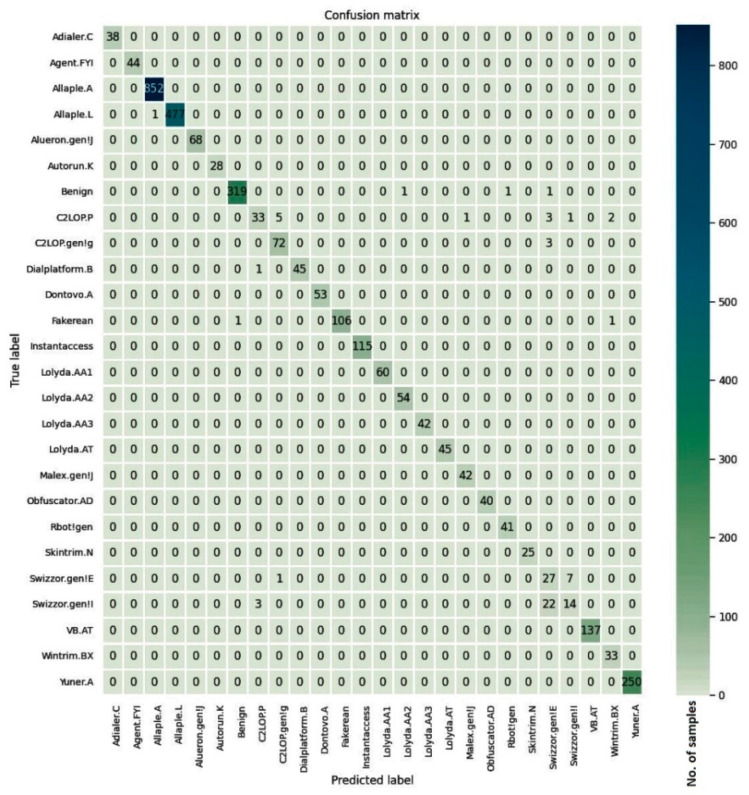
Confusion matrix for the Malimg dataset.

**Figure 9:**
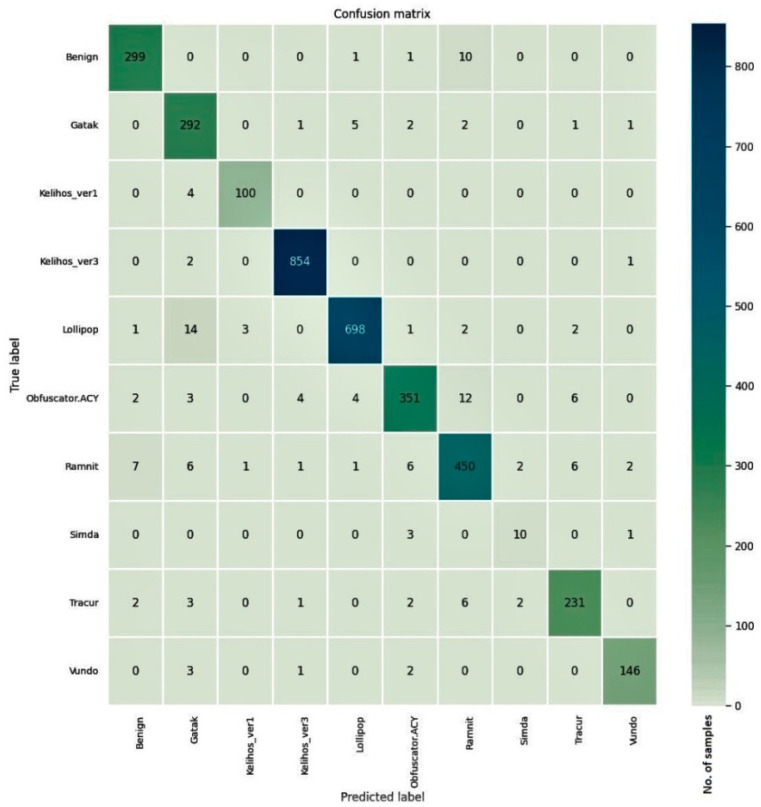
Confusion matrix for the BIG 2015 dataset.

**Figure 10:**
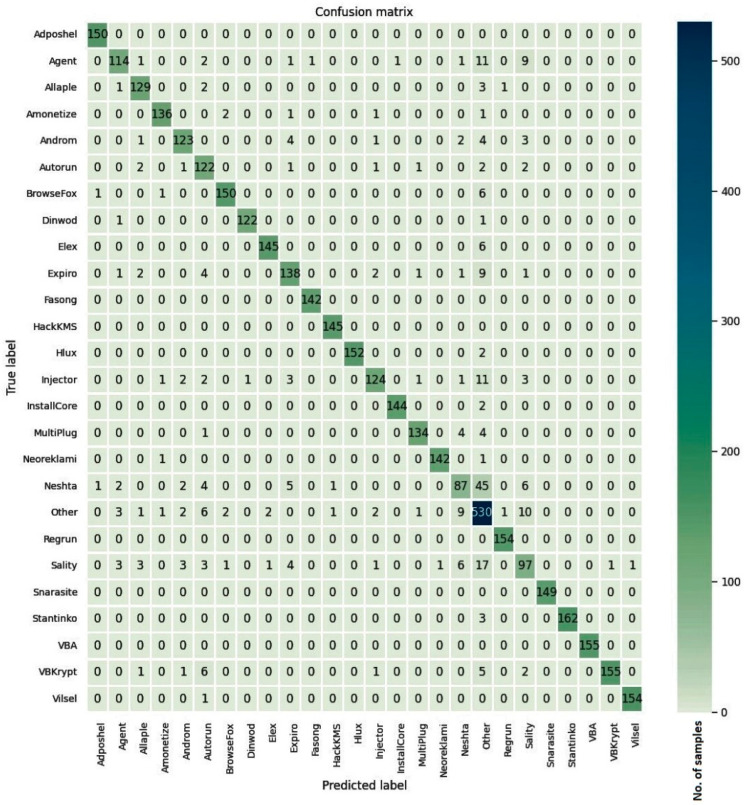
Confusion matrix for the MaleVis dataset.

The confusion matrices for the models trained on three malware datasets along with the cleanware class are given in Figure 8, Figure 9 and Figure 10. For the Malimg dataset with 26 classes, the confusion matrix is a 26×26 matrix with the columns representing the actual class and the rows indicating the predicted class. The diagonal elements show the number of correctly classified samples, where the predicted class matches the actual class. The off-diagonal elements represent misclassified samples. The diagonal elements for all three datasets show higher values compared to the off-diagonal elements. Although the samples in the Simda class are fewer, most of the samples in that class were correctly classified by the proposed model.

**Figure 11:**
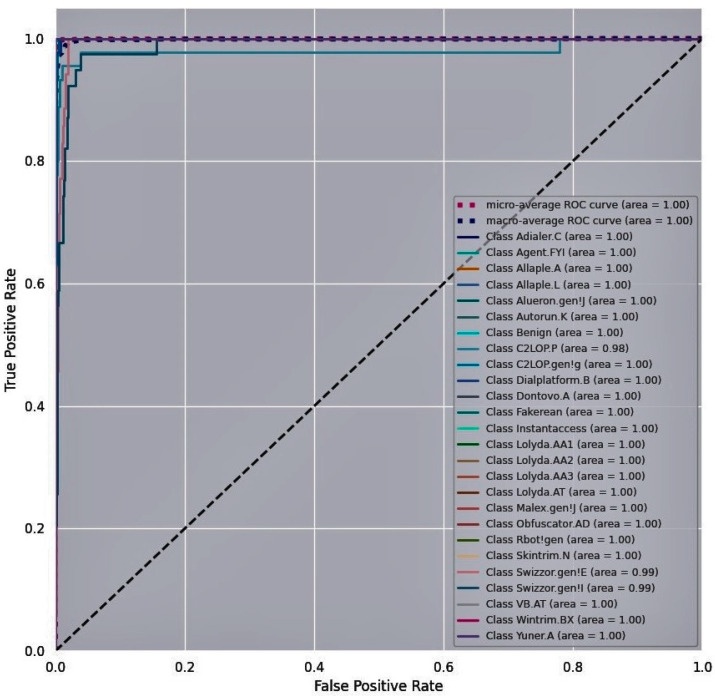
Receiver Operating Characteristic (ROC) curve for the Malimg dataset.

**Figure 12:**
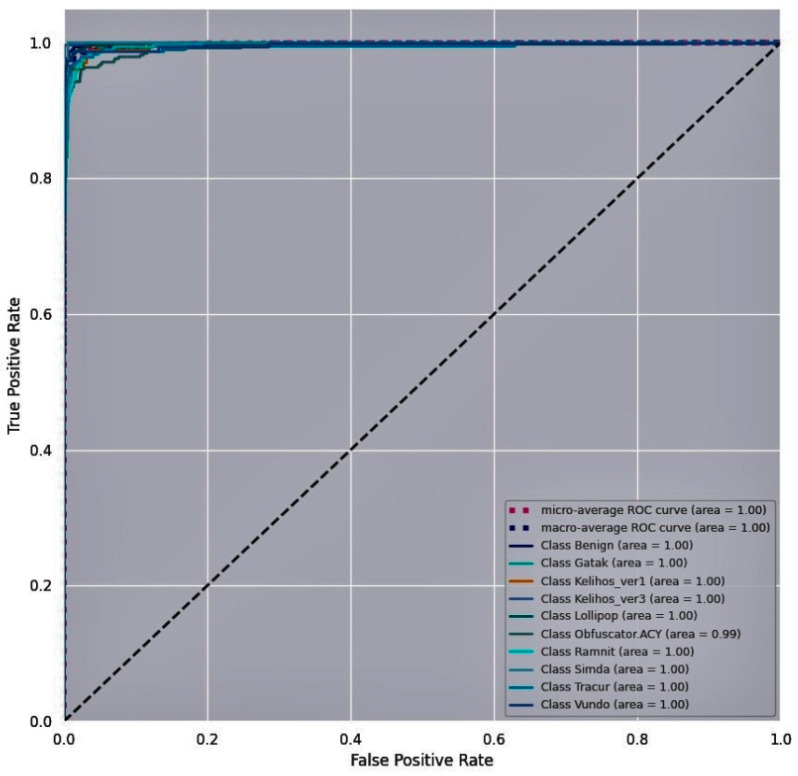
ROC curve for the BIG 2015 dataset.

**Figure 13:**
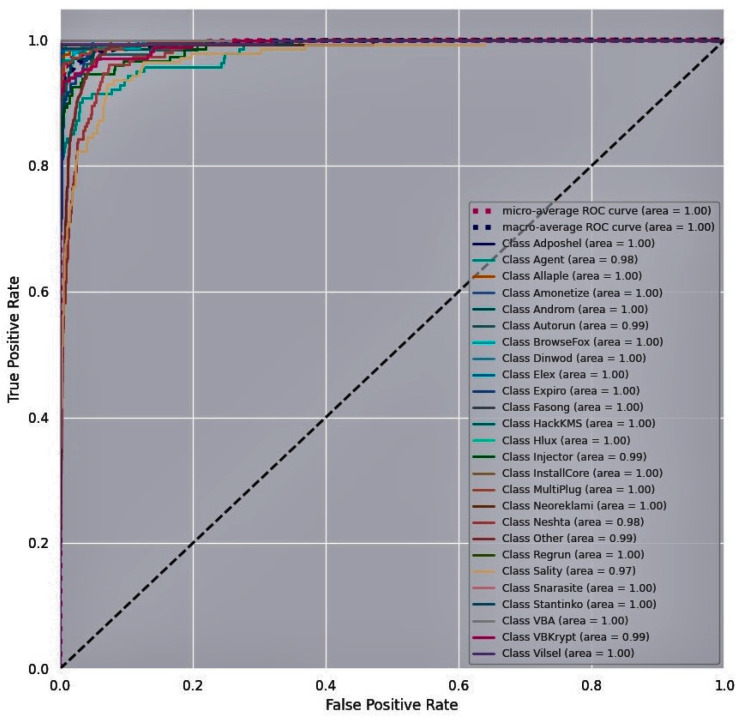
ROC curve for the MaleVis dataset.

A Receiver Operating Characteristic (ROC) curve is a plot of the True Positive Rate (TPR) vs. False Positive Rate (FPR) at different classification thresholds to examine the performance of the proposed malware detection model. Figure 11, Figure 12 and Figure 13 show the ROC curves for the proposed model obtained for the three training malware datasets. The N number of ROC curves corresponding to the N number of classes are seen in Figure 11, Figure 12 and Figure 13. For instance, the Malimg dataset includes 26 classes. The graph shows 26 ROC curves, with the first curve representing the first class that is classified against the other 25 classes, the next ROC curve representing the second class that is classified against the rest of the classes, and so on. TPR is approximately one and FPR is close to zero on the curves for each class against every other class. The area under the curve is higher for all the classes on the Malimg and BIG2015 malware datasets compared to the area under the curve for the MaleVis dataset. This indicates the outperforming efficiency of the proposed DenseNet-based malware detection model.

The proposed malware detection system would be effective and can produce advanced results, as shown in Table 7. Any new malware that resembles these families of malware will also be detected with the same accuracy because of the generalization property of the proposed model. If the new malware is completely unseen, i.e., a zero-day malware attack, the proposed system may fail to detect it. Therefore, if such zero-day attacks accumulate, then the performance of the proposed model could fall, but a false alarm may indicate that the model needs to be retrained. Therefore, the model will be retuned with new samples and the performance will be tuned such that the model will detect malware that has already been trained as well as newly seen malware, almost similar to a top-up of the training set. As a result, the proposed model would be able to keep up with malware evolution over time and to understand anti-malware evasion techniques.

The experiments were conducted for binary classification (malware or cleanware) with the Malimg, BIG2015, and MaleVis datasets. For each of the three datasets, 1000 samples were picked and included in the malware class, while the other class contained 1043 cleanware samples. The results were taken to assess the performance of the proposed DenseNet-based malware detection system for the three binary datasets. The accuracy for the BIG2015 binary dataset shows a higher detection accuracy of 97.72% compared to the other datasets. The accuracy for the Malimg binary dataset is 97.55%, and the accuracy for the MaleVis binary dataset is 96.81%. The other metrics such as precision, recall, and f1score are similarly higher for BIG2015 than for the other two binary datasets.

## 5. Conclusions

We proposed an efficient malware detection and classification technique that combines malware visualization and a pretrained DenseNet model with a reweighted categorical cross-entropy loss criterion. The performance of the proposed DenseNet-based malware detection approach was evaluated on four malware datasets, and its superiority over other models was analyzed.

The proposed model achieved a better classification accuracy of 98.23% for the Malimg dataset, of 98.46% for the BIG 2015 dataset, and of 98.21% for the MaleVis dataset, which is higher than the other methods explored. The accuracy of the unseen dataset that has not been trained by the proposed model achieves an accuracy of 89.48%.

The proposed model correctly identified most of the obfuscated malware samples, proving its resiliency towards malware mitigation methods. The proposed solution does not require execution or unpacking of the packed executables. The experiment results demonstrate that, even though the training set is imbalanced, our technique can effectively and efficiently classify malware samples to their corresponding families. The proposed detection system shows high accuracy and time performance that is comparable with conventional solutions based on machine learning while eliminating the manual feature engineering stage.

In the future, we will concentrate on the reduction of false negatives to achieve an optimal solution.

## Figures and Tables

**Table 1:** Various families in the malware datasets.

Datasets	Family Name
Malimg [10]	Yuner.A, Wintrim.BX, VB.AT, Swizzor.gen!E, Skintrim.N, Rbot!gen, Obfuscator.AD, Malex.gen!J, Lolyda.AT, Lolyda.AA3, Lolyda.AA2, Lolyda.AA1, Instantaccess, Fakerean, Dontovo.A, Dialplatform.B, C2LOP.P, C2LOP.gen!g, Autorun.K, Alueron.gen!J, Allaple.L, Allaple.A, Agent.FYI, Adialer.C
BIG 2015 [11]	Vundo, Tracur, Simda, Ramnit, Obfuscator.ACY, Lollipop, Kelihos_ver3, Kelihos_ver1, Gatak
MaleVis [12]	Vilsel, VBKrypt, VBA/Hilium.A, Stantinko, Snarasite.D!tr, Sality, Regrun.A, Neshta, Neoreklami, MultiPlug, InstallCore.C, Injector, Hlux!IK, HackKMS.A, Fasong, Expiro-H, Elex, Dinwod!rfn, BrowseFox, AutoRun-PU, Androm, Amonetize, Allaple.A, Agent-fyi, Adposhel
Malicia [16]	Zeroaccess, Zbot, Winwebsec, Smarthdd, Securityshield Harebot, Cridex, Cleaman

**Table 2:** Comparison of machine learning-based methods with the proposed method for the three training datasets. The best values are emphasized in bold.

Models	Malimg Dataset				BIG2015 Dataset				MaleVis Dataset			
	Acc (%)	Pr	Re	F-Score	Acc (%)	Pr	Re	F-Score	Acc (%)	Pr	Re	F-Score
KNN	82.4	0.8146	0.8233	0.8189	85.28	0.8614	0.8464	0.8538	84.36	0.8531	0.8357	0.8443
LR	69.2	0.6955	0.668	0.6815	62.59	0.6421	0.6235	0.6327	66.47	0.6686	0.6575	0.6630
SVM	75.1	0.7459	0.7534	0.7496	89.25	0.9042	0.8846	0.8943	88.38	0.8779	0.8748	0.8763
NB	56.25	0.5678	0.5547	0.5612	52.14	0.5158	0.5223	0.5190	55.62	0.5622	0.5474	0.5547
DT	88.47	0.8798	0.8721	0.8759	86.41	0.8632	0.8575	0.8603	87.35	0.8787	0.8663	0.8725
RF	90.75	0.9127	0.8963	0.9044	91.22	0.9179	0.9064	0.9121	90.28	0.8985	0.9055	0.9020
Adaboost	74.36	0.7463	0.7286	0.7373	83.68	0.8512	0.8244	0.8376	76.44	0.7564	0.7652	0.7608
**Proposed**	**98.23**	**0.9778**	**0.9792**	**0.9785**	**98.46**	**0.9858**	**0.9784**	**0.9821**	**98.21**	**0.9856**	**0.9774**	**0.9815**

**Table 3:** Comparison of deep learning-based methods with the proposed method for the three training datasets. The best values are emphasized in bold.

Models	Malimg Dataset				BIG 2015 Dataset				MaleVis Dataset			
	Acc (%)	Pr	Re	F-Score	Acc (%)	Pr	Re	F-Score	Acc (%)	Pr	Re	F-Score
CNN	97.59	0.9761	0.9748	0.9754	95.67	0.9573	0.9570	0.9571	94.38	0.9441	0.9438	0.9439
VGG16	97.44	0.9754	0.9742	0.9748	88.61	0.8872	0.8861	0.8866	96.18	0.9644	0.9576	0.9610
VGG19	97.51	0.9765	0.9753	0.9759	88.82	0.8886	0.8879	0.8882	96.27	0.9637	0.9627	0.9632
Inception-v3	97.65	0.9870	0.9864	0.9867	93.29	0.9336	0.9328	0.9332	95.32	0.9568	0.9499	0.9533
Resnet-50	97.68	0.9761	0.9768	0.9764	88.52	0.8868	0.8852	0.8860	90.36	0.9063	0.8994	0.9028
Xception	98.03	0.9796	0.9803	0.9799	96.78	0.9680	0.9673	0.9676	97.49	0.9757	0.9738	0.9747
DenseNet-121	98.15	0.9808	0.9814	0.9811	96.77	0.9672	0.9675	0.9673	95.27	0.9532	0.9515	0.9523
**Proposed**	**98.23**	**0.9778**	**0.9792**	**0.9785**	**98.46**	**0.9858**	**0.9784**	**0.9821**	**98.21**	**0.9856**	**0.9774**	**0.9815**

**Table 4:** Comparison of the proposed model with the Machine Learning (ML) and Deep Learning (DL) models for the Malicia (unseen) dataset. The best values are emphasized in bold.

Methods		Acc (%)	Pr	Re	F-Score
ML Methods	KNN	76.75	0.7753	0.7618	0.7685
	LR	56.33	0.5779	0.5612	0.5694
	SVM	80.33	0.8138	0.7961	0.8049
	Naïve Bayes	46.93	0.4642	0.4701	0.4671
	Decision Tree	77.77	0.7769	0.7718	0.7743
	Random Forest	82.10	0.8261	0.8158	0.8209
	Adaboost	75.31	0.7661	0.742	0.7538
DL Methods	CNN	71.42	0.722	0.7061	0.7139
	VGG16	77.66	0.7817	0.7765	0.7791
	VGG19	82.92	0.8288	0.827	0.8279
	Inception-v3	83.7	0.8358	0.825	0.8304
	Resnet-50	82.52	0.8312	0.8062	0.8185
	Densenet-121	83.02	0.8261	0.8186	0.8224
	Xception	83.02	0.8261	0.8186	0.8224
**Proposed Method**		**89.48**	**0.8936**	**0.8922**	**0.8929**

**Table 5:** Comparison of deep learning-based malware detection models based on computational time. The best values are emphasized in bold.

Models	Training Time (in sec)			Testing Time (in sec)		
	Malimg	BIG 2015	MaleVis	Malimg	BIG 2015	MaleVis
CNN	6140	4406	10946	7.82	8.14	8.58
VGG16	5174	3652	12721	6.67	6.84	7.04
VGG19	5363	3870	15144	6.35	6.61	6.74
Inception-v3	5604	4146	11379	5.89	6.08	6.36
Resnet-50	6097	4712	8861	7.36	8.12	8.58
Densenet-121	6574	5259	8328	8.48	8.70	8.96
Xception	5674	4226	10448	5.08	5.53	6.36
**Proposed**	**1941**	**2237**	**2351**	**4.36**	**4.49**	**5.09**

**Table 6:** Comparison of existing works with the proposed method for the four malware datasets. The best values are emphasized in bold.

Methods	Malimg Dataset				BIG 2015 Dataset				MaleVis Dataset				Malicia Dataset			
	Acc (%)	Pr	Re	F-Score	Acc (%)	Pr	Re	F-Score	Acc (%)	Pr	Re	F-Score	Acc (%)	Pr	Re	F-Score
Nataraj et al. [10]	97.18	0.9657	0.9685	0.9671	96.48	0.9646	0.9544	0.9595	91.69	0.9236	0.8958	0.9095	85.26	0.8493	0.8520	0.8506
Roseline et al. [58]	**98.65**	**0.9886**	**0.9863**	**0.9874**	97.2	0.9761	0.9679	0.9720	97.43	0.9753	0.9732	0.9742	86.45	0.8615	0.8636	0.8625
Cui et al. [54]	94.5	0.9464	0.9431	0.9447	93.4	0.9328	0.9354	0.9341	92.13	0.9209	0.9189	0.9199	80.17	0.7894	0.8008	0.7951
Agarap et al. [55]	84.92	0.8547	0.8464	0.8505	80.51	0.8135	0.7986	0.8060	79.36	0.8022	0.7845	0.7933	72.05	0.7195	0.7200	0.7197
Vinayakumar et al. [59]	96.3	0.963	0.9582	0.9606	91.27	0.9221	0.9132	0.9176	86.29	0.8685	0.8628	0.8656	84.63	0.8433	0.8426	0.8429
Luo et al. [60]	93.72	0.9413	0.9254	0.9333	93.57	0.9447	0.9268	0.9357	92.24	0.9179	0.9096	0.9137	82.54	0.8227	0.8235	0.8231
Singh [31]	96.08	0.9576	0.9616	0.9596	94.24	0.9423	0.9289	0.9356	93	0.9287	0.9167	0.9227	84.28	0.8384	0.8469	0.8426
**Proposed**	98.23	0.9778	0.9792	0.9785	**98.46**	**0.9858**	**0.9784**	**0.9821**	**98.21**	**0.9856**	**0.9774**	**0.9815**	**89.48**	**0.8936**	**0.8922**	**0.8929**

**Table 7:** Performance of the proposed method for three malware binary classification datasets.

Performance Metrics	Malimg Dataset	BIG2015 Dataset	MaleVis Dataset
**Acc (%)**	97.55	97.72	96.81
**Pr**	0.9743	0.9756	0.9650
**Re**	0.9750	0.9748	0.9681
**F-score**	0.9746	0.9752	0.9665

## Data Availability

Data is available from the corresponding author upon reasonable request.

## References

[1] Jang-Jaccard J., Nepal S. (2014). A survey of emerging threats in cybersecurity. J. Comput. Syst. Sci..

[2] Amoroso E. (2018). Recent progress in software security. IEEE Softw..

[3] Drew J., Moore T., Hahsler M. Polymorphic malware detection using sequence classification methods. Proceedings of the 2016 IEEE Security and Privacy Workshops (SPW).

[4] Canfora G., Mercaldo F., Visaggio C.A., Di Notte P. (2014). Metamorphic Malware Detection Using Code Metrics. Inf. Secur. J. A Glob. Perspect..

[5] OKane P., Sezer S., McLaughlin K. (2011). Obfuscation The hidden malware. IEEE Secur. Priv..

[6] Kuraku S., Kalla D. (2020). Emotet Malware—A Banking Credentials Stealer. Iosr J. Comput. Eng..

[7] Celik R., Gezer A. (2019). Behavioral Analysis of Trickbot Banking Trojan with its New Tricks. Int. J. Technol. Eng. Stud..

[8] Islam R., Tian R., Batten L.M., Versteeg S. (2013). Classification of malware based on integrated static and dynamic features. J. Netw. Comput. Appl..

[9] Subairu S.O., Alhassan J., Misra S., Abayomi-Alli O., Ahuja R., Damasevicius R., Maskeliunas R. (2020). An experimental approach to unravel effects of malware on system network interface. Lecture Notes in Electrical Engineering.

[10] Nataraj L., Karthikeyan S., Jacob G., Manjunath B.S. Malware images. Proceedings of the 8th International Symposium on Visualization for Cyber Security—VizSec 11.

[11] Ronen R., Radu M., Feuerstein C., Yom-Tov E., Ahmadi M. (2018). Microsoft Malware Classification Challenge. arXiv.

[12] Bozkir A.S., Cankaya A.O., Aydos M. Utilization and Comparison of Convolutional Neural Networks in Malware Recognition. Proceedings of the 27th Signal Processing and Communications Applications Conference (SIU).

[13] Deng J., Dong W., Socher R., Li L., Li K., Li F. ImageNet: A large-scale hierarchical image database. Proceedings of the 2009 IEEE Computer Society Conference on Computer Vision and Pattern Recognition (CVPR 2009).

[14] Huang G., Liu Z., Van Der Maaten L., Weinberger K.Q. Densely connected convolutional networks. Proceedings of the IEEE Conference on Computer Vision and Pattern Recognition.

[15] Tensorflow. www.tensorflow.org.

[16] Nappa A., Rafique M.Z., Caballero J. (2015). The MALICIA dataset identification and analysis of drive-by download operations. Int. J. Inf. Secur..

[17] Souri A., Hosseini R. (2018). A state-of-the-art survey of malware detection approaches using data mining techniques. Hum. Cent. Comput. Inf. Sci..

[18] Odusami M., Abayomi-Alli O., Misra S., Shobayo O., Damasevicius R., Maskeliunas R. (2018). Android malware detection: A survey. Applied Informatics. ICAI 2018. Communications in Computer and Information Science.

[19] Santos I., Brezo F., Ugarte-Pedrero X., Bringas P.G. (2013). Opcode sequences as representation of executables for data-mining-based unknown malware detection. Inf. Sci..

[20] Tian R., Batten L.M., Versteeg S.C. Function length as a tool for malware classification. Proceedings of the 3rd International Conference on Malicious and Unwanted Software (MALWARE).

[21] Kong D., Yan G. Discriminant malware distance learning on structural information for automated malware classification. Proceedings of the 19th ACM SIGKDD International Conference on Knowledge Discovery and Data Mining.

[22] Wadkar M., Di Troia F., Stamp M. (2020). Detecting malware evolution using support vector machines. Expert Syst. Appl..

[23] Schultz M.G., Eskin E., Zadok F., Stolfo S.J. Data mining methods for detection of new malicious executables. Proceedings of the 2001 IEEE Symposium on Security and Privacy (SP 2001).

[24] Roseline S.A., Geetha S. Intelligent Malware Detection using Oblique Random Forest Paradigm. Proceedings of the International Conference on Advances in Computing, Communications and Informatics (ICACCI).

[25] Kim H., Kim J., Kim Y., Kim I., Kim K.J., Kim H. (2019). Improvement of malware detection and classification using API call sequence alignment and visualization. Clust. Comput..

[26] Imran M., Afzal M.T., Qadir M.A. Similarity-based malware classification using hidden Markov model. Proceedings of the Fourth International Conference on Cyber Security, Cyber Warfare, and Digital Forensic (CyberSec).

[27] Kolter J.Z., Maloof M.A. (2006). Learning to detect and classify malicious executables in the wild. J. Mach. Learn. Res..

[28] Rieck K., Trinius P., Willems C., Holz T. (2011). Automatic analysis of malware behavior using machine learning. J. Comput. Secur..

[29] Roseline S.A., Hari G., Geetha S., Krishnamurthy R. Vision-Based Malware Detection and Classification Using Lightweight Deep Learning Paradigm. Proceedings of the International Conference on Computer Vision and Image Processing.

[30] Roseline S.A., Sasisri A.D., Geetha S., Balasubramanian C. Towards Efficient Malware Detection and Classification using Multilayered Random Forest Ensemble Technique. Proceedings of the 2019 International Carnahan Conference on Security Technology (ICCST).

[31] Singh A., Handa A., Kumar N., Shukla S.K. Malware classification using image representation. Proceedings of the International Symposium on Cyber Security Cryptography and Machine Learning.

[32] Shiva Darshan S.L., Jaidhar C.D. (2019). Windows malware detector using convolutional neural network based on visualization images. IEEE Trans. Emerg. Top. Comput..

[33] Vasan D., Alazab M., Wassan S., Safaei B., Zheng Q. (2020). Image-based malware classification using ensemble of CNN architectures (IMCEC). Comput. Secur..

[34] Zhang J., Qin Z., Yin H., Ou L., Xiao S., Hu Y. Malware variant detection using opcode image recognition with small training sets. Proceedings of the 25th International Conference on Computer Communication and Networks (ICCCN).

[35] Han K., Kang B., Im E.G. (2014). Malware analysis using visualized image matrices. Sci. World J..

[36] Yan H., Zhou H., Zhang H. (2018). Automatic malware classification via PRICoLBP. Chin. J. Electron..

[37] Conti G., Dean E., Sinda M., Sangster B. (2008). Visual reverse engineering of binary and data files. Proceedings of the International Workshop on Visualization for Computer Security.

[38] Kancherla K., Mukkamala S. Image visualization based malware detection. Proceedings of the 2013 IEEE Symposium on Computational Intelligence in Cyber Security (CICS).

[39] Liu L., Wang B. Malware classification using gray-scale images and ensemble learning. Proceedings of the 3rd International Conference on Systems and Informatics (ICSAI).

[40] Fu J., Xue J., Wang Y., Liu Z., Shan C. (2018). Malware visualization for fine-grained classification. IEEE Access.

[41] Nisa M., Shah J.H., Kanwal S., Raza M., Khan M.A., Damaševičius R., Blažauskas T. (2020). Hybrid malware classification method using segmentation-based fractal texture analysis and deep convolution neural network features. Appl. Sci..

[42] Azab A., Khasawneh M. (2020). MSIC Malware spectrogram image classification. IEEE Access.

[43] Ding Y., Zhang X., Hu J., Xu W. (2020). Android malware detection method based on bytecode image. J. Ambient Intell. Humaniz. Comput..

[44] Mahdavifar S., Ghorbani A.A. (2020). DeNNeS Deep embedded neural network expert system for detecting cyber attacks. Neural Comput. Appl..

[45] Naeem H., Ullah F., Naeem M.R., Khalid S., Vasan D., Jabbar S., Saeed S. (2020). Malware detection in industrial internet of things based on hybrid image visualization and deep learning model. Ad Hoc Netw..

[46] Singh J., Thakur D., Ali F., Gera T., Kwak K.S. (2020). Deep feature extraction and classification of android malware images. Sensors.

[47] Sun G., Qian Q. (2021). Deep learning and visualization for identifying malware families. IEEE Trans. Dependable Secur. Comput..

[48] LeCun Y., Bengio Y., Hinton G. (2015). Deep learning. Nature.

[49] Zhong W., Gu F. (2019). A multi-level deep learning system for malware detection. Expert Syst. Appl..

[50] Ni S., Qian Q., Zhang R. (2018). Malware identification using visualization images and deep learning. Comput. Secur..

[51] Yong B., Wei W., Li K., Shen J., Zhou Q., Wozniak M., Połap D., Damaševičius R. (2020). Ensemble machine learning approaches for webshell detection in internet of things environments. Trans. Emerg. Telecommun. Technol..

[52] Azeez N.A., Odufuwa O.E., Misra S., Oluranti J., Damaševičius R. (2021). Windows PE Malware Detection Using Ensemble Learning. Informatics.

[53] Damaševičius R., Venčkauskas A., Toldinas J., Grigaliūnas Š. (2021). Ensemble-Based Classification Using Neural Networks and Machine Learning Models for Windows PE Malware Detection. Electronics.

[54] Cui Z., Xue F., Cai X., Cao Y., Wang G.G., Chen J. (2018). Detection of malicious code variants based on deep learning. IEEE Trans. Ind. Inform..

[55] Agarap A.F., Pepito F.J.H. (2017). Towards building an intelligent anti-malware system a deep learning approach using support vector machine (SVM) for malware classification. arXiv.

[56] Cui Y., Jia M., Lin T.Y., Song Y., Belongie S. Class-balanced loss based on effective number of samples. Proceedings of the IEEE Conference on Computer Vision and Pattern Recognition.

[57] Kingma D.P., Ba J. (2014). Adam: A method for stochastic optimization. arXiv.

[58] Roseline S.A., Geetha S., Kadry S., Nam Y. (2020). Intelligent Vision-based Malware Detection and Classification using Deep Random Forest Paradigm. IEEE Access.

[59] Vinayakumar R., Alazab M., Soman K.P., Poornachandran P., Venkatraman S. (2019). Robust intelligent malware detection using deep learning. IEEE Access.

[60] Luo J.S., Lo D.C.T. Binary malware image classification using machine learning with local binary pattern. Proceedings of the IEEE International Conference on Big Data (Big Data).

